# Detecting atrial fibrillation in the polysomnography-derived electrocardiogram: a software validation study

**DOI:** 10.1007/s11325-023-02779-3

**Published:** 2023-01-21

**Authors:** Julia van Kempen, Christian Glatz, Julian Wolfes, Gerrit Frommeyer, Matthias Boentert

**Affiliations:** 1 Department of Neurology with Institute of Translational Neurology, Münster University Hospital (UKM), Münster, Germany; 2 Department of Cardiology II - Electrophysiology, Münster University Hospital (UKM), Münster, Germany; 3 Department of Medicine, UKM Marienhospital Steinfurt, Steinfurt, Germany

**Keywords:** Electrocardiogram, Polysomnography, Atrial fibrillation, Automated ECG analysis

## Abstract

**Purpose:**

The present study validated a software-based electrocardiogram (ECG) analysis tool for detection of atrial fibrillation (AF) and risk for AF using polysomnography (PSG)-derived ECG recordings.

**Methods:**

The Stroke Risk Analysis® (SRA®) software was applied to 3-channel ECG tracings from diagnostic PSG performed in enrolled subjects including a subgroup of subjects with previously documented AF. No subjects used positive airway pressure therapy. All ECG recordings were visually analyzed by a blinded cardiologist.

**Results:**

Of subjects enrolled in the study, 93 had previously documented AF and 178 of 186 had an ECG that could be analyzed by either method. In subjects with known history of AF, automated analysis using SRA® classified 47 out of 87 ECG as either manifest AF or showing increased risk for paroxysmal AF (PAF) by SRA® (sensitivity 0.54, specificity 0.86). On visual analysis, 36/87 ECG showed manifest AF and 51/87 showed sinus rhythm. Among the latter subgroup, an increased risk for PAF was ascribed by SRA® in 11 cases (sensitivity 0.22, specificity 0.78) and by expert visual analysis in 5 cases (sensitivity 0.1, specificity 0.90). Among 36/178 ECG with manifest AF on visual analysis, 33 were correctly identified by the SRA® software (sensitivity and specificity 0.92).

**Conclusion:**

Sleep studies provide a valuable source of ECG recordings that can be easily subjected to software-based analysis in order to identify manifest AF and automatically assess the risk of PAF. For optimal evaluability of data, multiple channel ECG tracings are desirable. For assessment of PAF risk, the SRA® analysis probably excels visual analysis, but sensitivity of both methods is low, reflecting that repeated ECG recording remains essential.

**Supplementary Information:**

The online version contains supplementary material available at 10.1007/s11325-023-02779-3.

## Introduction

About 20% of ischemic strokes are caused by heart disease and atrial fibrillation (AF), in particular [1]. The prevalence of AF has been reported to be 0.51% worldwide and 1–4% in industrialized countries [2, 3]. Stroke etiology cannot be unambigously identified in more than 30% of cases (“cryptogenic stroke”) [4], likely including subjects with paroxysmal AF (PAF) that may often go undetected [5–7]. Cardio-embolic stroke is frequently severe due to occlusion of large cerebral vessels [8]. As ischemic stroke due to AF can be effectively prevented by oral anticoagulation, reliable detection of AF is essential for identifying subjects at risk. However, detection of PAF is known to be delayed in many cases [9], and detection rates strongly depend on the applied type of cardiac monitoring. Frequently, PAF is missed by standard 12-channel electrocardiography (ECG) and 24-h Holter ECG recordings. Repeated or extended 24-h Holter ECG recording substantially enhances PAF detection rate [10–12]. Insertable or implantable cardiac monitors have been shown to identify even more subjects with PAF [13]. Lastly, several methods for automated ECG analysis have been developed [14–16]. Among these, the Stroke Risk Analysis (SRA®) software utilizes a computational algorithm identifying either manifest AF or yielding a graded score that classifies the risk of AF [17].

Among many other conditions, sleep-disordered breathing (SDB) has been identified as an independent risk factor for AF [18, 19]. For diagnostic purposes, cardiorespiratory polygraphy and polysomnography (PSG) are widely used if SDB is suspected, both including ECG recordings. Routine sleep studies are usually conducted over a 6–8-h period, providing an ECG recording of the same duration that can potentially be used for automated ECG analysis and individual risk assessment. However, the SRA® technology has not yet been applied to ECG recordings derived from overnight sleep studies. Therefore, this study investigated whether the SRA® software correctly identifies subjects with manifest AF and accurately classifies subjects with previously documented PAF as being at risk based on a 3-lead polysomnographic ECG.

## Methods

**Fig. 1:**
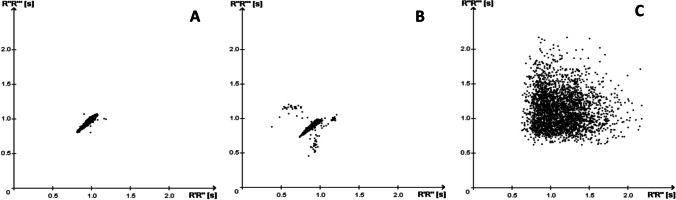
Example of Lorenz plots derived from SRA® analysis. **a,** Patient with sinus rhythm and regular R-R intervals, **b,** patient with sinus rhythm and irregular R-R intervals classified as “increased risk of AF”, **c,** patient with manifest AF. SRA®, stroke risk analysis, AF, atrial fibrillation

This retrospective study was approved by the local ethics authority (Ethikkommission der Ärztekammer Westfalen-Lippe und der Medizinischen Fakultät der WWU Münster, Az. 2019–238-f-S). PSG recordings were extracted from the sleep laboratory database at Münster University Hospital (10/2009–02/2019). Inclusion criteria comprised age > 18 years, diagnostic PSG (i.e., no use of positive airway pressure therapy) and total sleep time > 6 h. Subjects wearing a cardiac pacemaker were excluded. Patients with known chronic AF or PAF at the time of diagnostic sleep studies were identified based on pre-existing information derived from hospital records (ICD-10 codes I48.0, I48.1, and I48.2). Subjects in whom manifest AF had been identified on visual evaluation of the PSG-derived ECG were also included in this subgroup. Medical records and PSG data of control subjects were extracted from the same clinical database. Control subjects had no known AF, did not use positive airway pressure therapy, and were matched for age (± 3 years) and gender. Diagnostic PSGs were performed for evaluation of sleep-related breathing, insomnia, parasomnia, or hypersomnolence. Cardiorespiratory PSG (Nihon Kohden, Rosbach, Germany) was conducted and validated following standard recommendations. ECG recordings comprised three leads and were extracted from the PSG record in European Data Format (EDF) for SRA® analysis. SRA® technology uses non-linear and linear computational algorithms to evaluate the dynamics of R-R interval variations. Increased or irregular R-R intervals are known to occur more frequently in subjects with PAF even in periods with sinus rhythm. SRA® technology classifies QRS complexes as either normal or ventricular based on morphological features. For further mathematical analysis, an algorithm is applied that was trained in a neural network setup for which a large number of ECG datasets from subjects with and without PAF were used [15]. Finally, the risk for AF is classified according to four categories: 0, no increased risk for paroxysmal AF (normal); 1, increased risk for PAF; 2, manifest AF; *x*, not evaluable. The results are displayed using a Lorenz plot that depicts successive R-R intervals in an alternating manner on the *x*-axis and *y*-axis of a Cartesian coordinate system (Fig. 1). Finally, all ECG recordings were visually and independently analyzed by an expert cardiologist (G. F.) who was blinded for both group affiliation of ECG recordings and results of SRA® analysis. Visually analyzed ECG recordings were also grouped according to the above four categories (i.e., normal, increased risk of AF, manifest AF, not evaluable). Increased risk of AF was thought to be reflected by occurrence of premature supraventricular beats and short atrial runs that have been shown to predict future AF (or PAF, respectively) in patients with cryptogenic stroke [20].

## Results

In total, PSG recordings from 186 individuals (56 women, mean age 65, standard deviation 12) were used for data analysis. The patient cohort comprised 93 subjects with documented AF and 93 controls. All ECG tracings could successfully be extracted for both software-based and independent visual analysis. Among the entire cohort, the number of recordings that were considered technically unevaluable by either SRA® or visual analysis was 4 (2.1%) and 7 (3.7%), respectively. Finally, 178 ECG tracings were analyzable by both methods.

### SRA® analysis

By SRA® analysis, 35/178 (19.7%) ECGs were classified as manifest AF, 25/178 (14.0%) as “increased risk of AF,” and 118/178 (66.3%) as unremarkable. Among 89 evaluable ECG tracings from patients with documented AF, classification was as follows: 33 manifest AF, 14 increased risk, and 42 no increased risk of AF. Regarding the status “previously documented AF,” sensitivity and specificity of SRA® analysis were 0.54 and 0.86, respectively. Among controls (i.e., without previously documented AF), 11/93 patients were categorized as showing increased risk for PAF, and in 2/93 subjects, manifest AF was detected.

### Expert visual analysis

In 179 evaluable ECGs, visual analysis revealed manifest AF in 36 recordings which were all derived from patients with previously documented AF. Supraventricular extrasystoles (SVES), potentially indicating increased risk of AF [20], were observed in 5/88 patients with documented AF, and in 9/91 control subjects. In patients with previously documented AF (*n* = 88), sensitivity and specificity of visual ECG analysis for detection of either AF or an increased risk was 0.48 and 0.90 respectively.

### Matching of SRA® and visual analysis

**Fig. 2:**
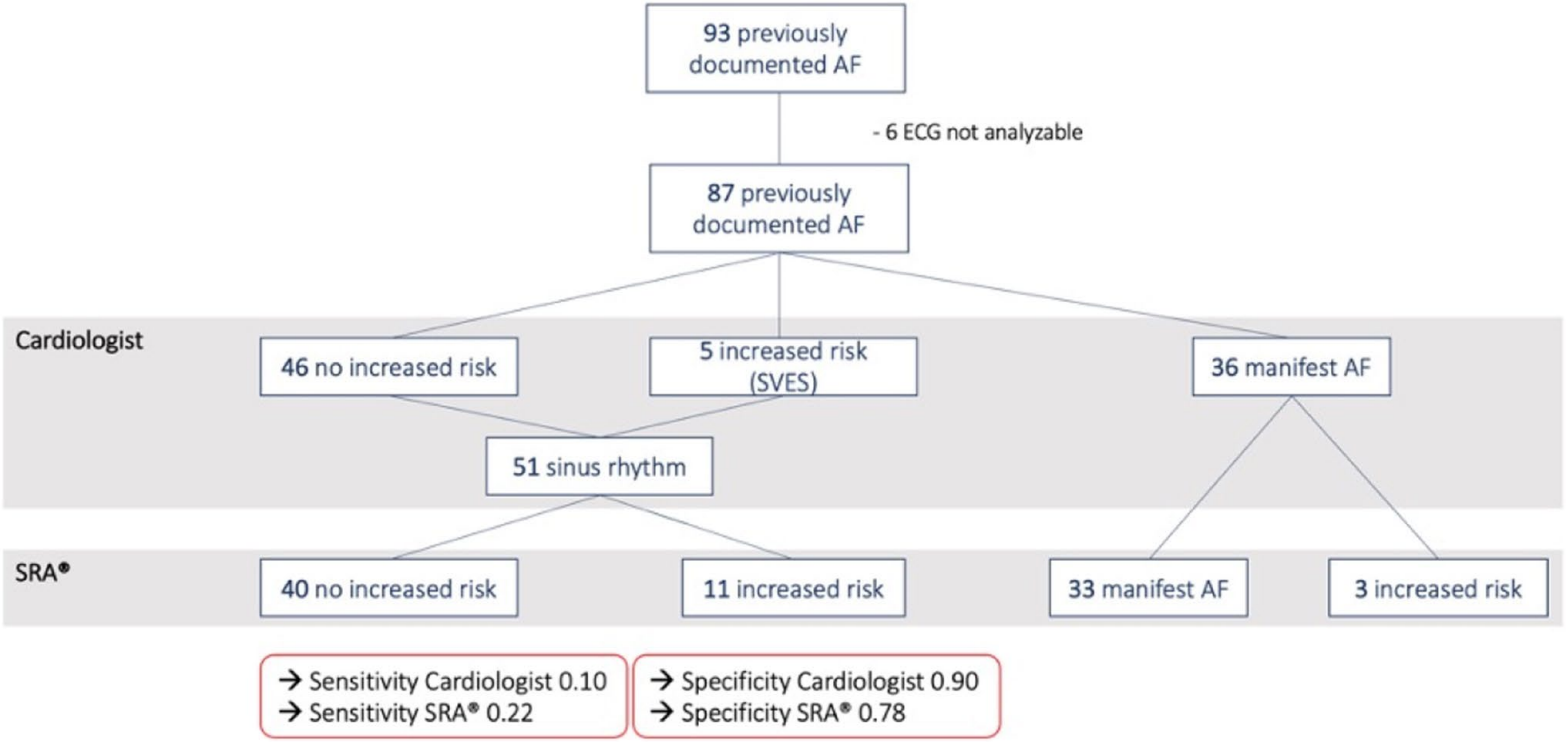
Software-based and visual ECG analysis in patients with previously documented AF

From 178 ECG recordings, 163 tracings were classified by the cardiologist and SRA® in consensus. In fifteen cases, ECG interpretation was discordant, including 3 ECG that were categorized as “increased risk of AF” by SRA® but were classified as manifest AF by visual analysis. In two cases, classification was manifest AF by SRA® but sinus rhythm with SVES by visual analysis. In ten ECG showing sinus rhythm, SRA® and visual analysis did not agree on risk assessment. On visual analysis, 36/87 ECG showed manifest AF and 51/87 showed sinus rhythm (Fig. 2). Among the latter subgroup, an increased risk for paroxysmal AF was ascribed by SRA® in 11 cases (sensitivity 0.22, specificity 0.78). The SRA® software correctly identified 33/36 ECGs that were classified as manifest AF by visual evaluation and categorized all remaining three recordings as “increased risk of AF.” Thus, none of these ECG traces were falsely classified as normal by automated analysis. With regard to the cardiologist’s interpretation, sensitivity and specificity of the SRA® analysis were both 0.92.

### Intercorrelation of AF and obstructive sleep apnea

Among 186 patients, 82 (44.1%) showed obstructive sleep apnea (OSA) on diagnostic PSG. Prevalence of OSA was 58.1% in patients with and 30.1% in subjects without previously documented AF (*p* < 0.001). Based on visual ECG classification, OSA was present in 32.0% of patients with sinus rhythm, 57.1% of those with increased risk of AF, and 81.1% of subjects with manifest AF (*p* < 0.001). Vice versa, manifest AF was present in 30/82 (36.5%) patients with newly diagnosed OSA compared to only 7/102 (6.7%) individuals without sleep-disordered breathing (*p* < 0.001).

## Discussion

The present study is the first to test both suitability and validity of the SRA® analysis software for automated evaluation of ECG recordings derived from overnight sleep studies. While it does not follow a prospective approach (e.g., by focusing on the prediction of future AF), the present work merely investigates whether the SRA® software is applicable to 3-channel PSG-derived ECG recordings. The study rationale is based on the assumption that sleep laboratory ECG data might be a valuable source for cardiovascular risk assessment in patients undergoing sleep studies. Sleep-disordered breathing (SDB) is highly prevalent in the aging population [21] and the most frequent cause for sleep laboratory evaluations. At the same time, it is a known risk factor for AF [18], rendering it reasonable to combine sleep studies with ECG analysis that is focused on either manifest AF or, using and automated approach, predictors of PAF risk. Many patients with SDB undergo repetitive evaluation of sleep once mask-based therapy has been established, increasing the number and cumulative duration of ECG recordings. As many PSG systems include more than one ECG channel, reliable tracings are available that can be used for automated ECG analysis.

This study confirms a substantial overlap (or coincidence) between OSA and AF. Prevalence of OSA was highest in patients with previously documented AF and with manifest AF, in particular. Based on expert visual analysis, OSA prevalence was intermediate in subjects showing sinus rhythm but ECG abnormalities that suggest an increased risk for PAF, indicating that the association between OSA and AF reflects a disease continuum. These findings are not pivotal for the present validation study but support previous work suggesting that SDB promotes AF risk and triggers episodes of manifest AF [22, 23].

The main result of this study is that SRA® analysis can technically be applied to ECG recordings that derive from overnight PSG. The proportion of tracings that cannot be automatically analysed is acceptably small, and with regard to detection of manifest AF, automated ECG analysis is not inferior to visual validation. Regarding prediction of PAF or PAF risk in patients with previously documented AF, test accuracy of the SRA® software is similar to visual ECG analysis. However, sensitivity of both methods is obviously limited and might be considered insufficient. This finding can be ascribed to several reasons. Firstly, duration of PSG-derived ECG recordings is still limited which likely causes a decrease in sensitivity as compared to multiple-day inpatient monitoring, repeated Holter ECG recordings, or long-term ECG tracing by means of implantable devices. Secondly, stratification of patients as “having documented AF” is possibly based on unreliable information that is derived from written medical records only that often cannot be verified. Thirdly, sensitivity of both automated and visual ECG analysis for detection of AF risk has also been shown to be limited in patients with cryptogenic stroke [20], indicating that a subset of patients with PAF cannot be identified by either method. As PSG-derived ECG recordings are technically suitable for automated analysis, longitudinal studies are desirable to investigate whether routine application of this method may help to prospectively identify patients at risk for PAF.

### Supplementary Information

Below is the link to the electronic supplementary material.

Supplementary file1 Overview of the study flow. AF, atrial fibrillation, SRA®, stroke risk analysis. (PPTX 425 KB)

## Data Availability

The datasets generated during and/or analyzed during the current study are available from the corresponding author on reasonable request.
