# Supplementary figures and images for: Detecting atrial fibrillation in the polysomnography-derived electrocardiogram: a software validation study
Source: Sleep Breath. 2023 Jan 21;27(5):1753–7. doi: 10.1007/s11325-023-02779-3 (PMC10539451; doi:10.1007/s11325-023-02779-3)

## Slide 1
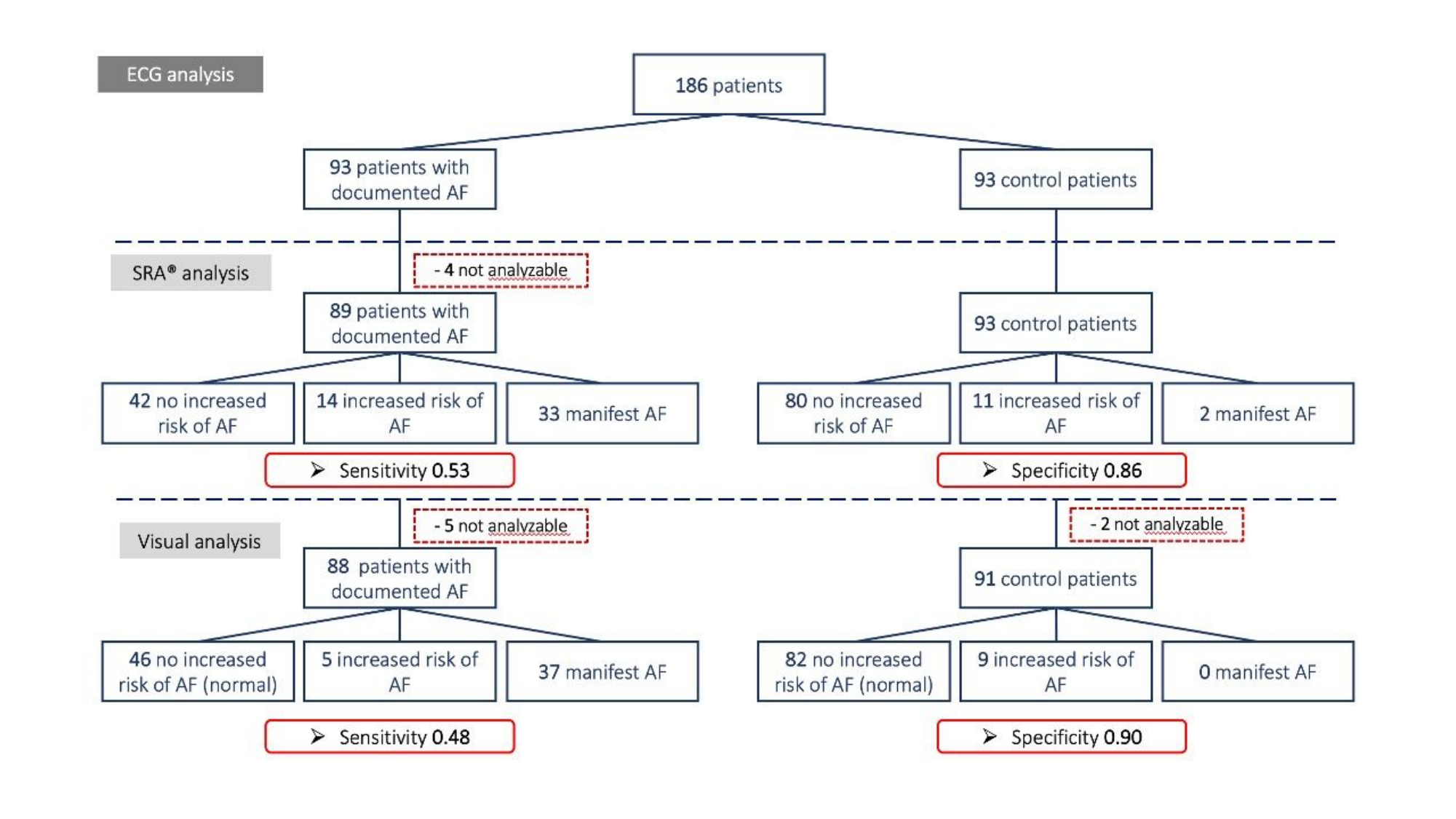

Supplement: Supplementary file 1 — Supplementary file1 Overview of the study flow. AF, atrial fibrillation, SRA®, stroke risk analysis. (PPTX 425 KB) [file 11325_2023_2779_MOESM1_ESM.pptx]
